# Effects of Physiological Signal Removal on Resting-State Functional MRI Metrics

**DOI:** 10.3390/brainsci13010008

**Published:** 2022-12-20

**Authors:** Uk-Su Choi, Yul-Wan Sung, Seiji Ogawa

**Affiliations:** 1 Medical Device Development Center, Daegu-Gyeongbuk Medical Innovation Foundation, Daegu 41061, Republic of Korea; 2 Kansei Fukushi Research Institute, Tohoku Fukushi University, Sendai 9893201, Japan

**Keywords:** functional MRI (fMRI), resting state fMRI (rs-fMRI), physiological signals, reliability, intra-session, intraclass correlation coefficient, functional connectivity, regional homogeneity, metric

## Abstract

Resting-state fMRIs (rs-fMRIs) have been widely used for investigation of diverse brain functions, including brain cognition. The rs-fMRI has easily elucidated rs-fMRI metrics, such as the fractional amplitude of low-frequency fluctuation (fALFF), regional homogeneity (ReHo), voxel-mirrored homotopic connectivity (VMHC), and degree centrality (DC). To increase the applicability of these metrics, higher reliability is required by reducing confounders that are not related to the functional connectivity signal. Many previous studies already demonstrated the effects of physiological artifact removal from rs-fMRI data, but few have evaluated the effect on rs-fMRI metrics. In this study, we examined the effect of physiological noise correction on the most common rs-fMRI metrics. We calculated the intraclass correlation coefficient of repeated measurements on parcellated brain areas by applying physiological noise correction based on the RETROICOR method. Then, we evaluated the correction effect for five rs-fMRI metrics for the whole brain: FC, fALFF, ReHo, VMHC, and DC. The correction effect depended not only on the brain region, but also on the metric. Among the five metrics, the reliability in terms of the mean value of all ROIs was significantly improved for FC, but it deteriorated for fALFF, with no significant differences for ReHo, VMHC, and DC. Therefore, the decision on whether to perform the physiological correction should be based on the type of metric used.

## 1. Introduction

Resting-state fMRI (rs-fMRI) signals come from intrinsic brain activities [1,2,3]. rs-fMRI has been applied to studies of brain disorders such as Alzheimer’s disease (AD), autism spectrum disorder (ASD), depression, etc., and normal sensory, higher-order cognitive, and higher-order social functions [4,5,6,7,8,9,10,11,12,13,14,15,16].

Contrary to the typical fMRI that uses tasks or stimulation, brain function studies with rs-fMRI require a metric to estimate brain activity. This requirement induced the development of metrics such as functional connectivity (FC) [17], the fractional amplitude of low-frequency fluctuation (fALFF) [18], regional homogeneity (ReHo) [19], voxel-mirrored homo-topic connectivity (VMHC) [20], degree centrality (DC) [21,22], etc. These metrics use a time series of brain rs-fMR images to evaluate different characteristics of brain activity. For example, FC reflects temporal coherence between brain areas. FC has been widely used to find intrinsic brain function such as the default mode network (DMN). This is a functional network produced from the correlation of rs-fMRI time series among the medial prefrontal cortex, posterior cingulate cortex/precuneus, and the angular gyrus known to be active during the rest condition, that is, when not executing any task [1,3]. The DMN is a crucial brain function network commonly used for identifying brain areas related to cognitive function [23], or for disorders such as AD and ASD [24,25,26]. 

Although most previous brain function studies were based on a population (a group of subjects), the requirement to identify brain disorders or function at an individual level requires improvements in the rs-fMRI (rs-fMRI more reliably reflects brain activity). Several factors affect rs-fMRI reliability; one of the major factors is physiological artifacts.

The rs-fMRI time series inevitably contains unwanted information or artifacts (signals not relevant to brain function) of a physiological origin, as well as interesting functional information. Two major physiological signal sources are cardiac pulsatory and respiration; these are difficult to remove. The cardiac rhythm affects the rs-fMRI through vessel expansion and the associated movement in nearby tissue compartments rather than blood oxygenation changes; meanwhile, respiration affects the rs-fMRI through changes in blood oxygenation. These physiological noises could explain a large fraction of fMRI variance in fMRIs related to both low- and high-frequency physiological fluctuations [27]. As a consequence, they act as noise sources that affect the metrics for evaluating rs-fMRI signals, and vary from person to person or from measurement to measurement. 

Previous studies claimed that removing physiological noises would be crucial to accurately quantify dynamic relationships within and across brain networks [28,29], since systemic hemodynamic fluctuations induced by heart rate changes can lead to artificial connectivity as neuronally-driven changes [30,31]. Their claim was supported by reports on the positive effects of physiological noise removal, such as improved temporal SNR (tSNR) and region specificity within the DMN which reduce apparent false positives. 

Rs-fMRI data correction—physiological noise removal—is achieved by the removal of time-locked cardiac and respiratory artifacts [32]. The most typically used correction method is to regress out cardiac and respiratory signals that are independently measured by the pulse and respiratory sensor from rs-fMRI signals at each voxel of the brain image. However, there are different connections from the physiological noise source to each voxel; the regression of physiological noise, regardless of the method, works differently at imaging voxels or areas of the brain because the mechanism representing the noise on the voxels or areas varies through the different vasculatures and tissue structures that surround them. 

Despite some reports on the positive effects of noise removal, others reported negative effects [33,34]; therefore, the question remains open. In addition, previous studies evaluated the effects of noise removal mostly on particular brain areas or networks [35]. Therefore, it is worthwhile to evaluate the applicability of this correction across the whole brain, especially analytically and quantitatively, with intra-session measurements. In this study, we examined the correction effect through the whole brain by evaluating the independent effect in each brain area where we applied the widely used correction method RETROICOR, present in fMRI analysis software packages such as FSL [36], SPM [37], and AFNI [38]. Particularly, we evaluated the intra-session reliability of the correction effects by repeating the measurement twice per session, with an interval of 2 or 3 days between sessions, for a total of six or eight sessions per subject for one month. The evaluation was performed for five typical rs-fMRI metrics of FC, fALFF, ReHo, VMHC, and DC that reflect different aspects of the rs-fMRI signal, and different dependences of rs-fMRI metrics on the physiological noise. For instance, fALFF is independent of characters between voxels such as correlation or coherence. ReHo is dependent on local coherence. FC is the correlation between ROIs. VMHC is the hemispheric coherence. DC represents connectivity, a second-order characteristic.

## 2. Materials and Methods

### 2.1. Subjects

This study involved three participants (all female, aged 20–23 years) who did not have a history of neurological disease or any other medical conditions. All participants were right-handed, and were registered in the same college. All participants provided written informed consent, and all experiments were approved by the Institutional Review Board of Tohoku Fukushi University.

### 2.2. MRI Acquisition

Two participants were scanned eight times and one participant six times, on different days, twice a week over one month, using a 3T investigational MRI scanner (Skyra-fit) with a default 20-channel head coil. Three-dimensional whole-brain high-resolution T1-weighted (T1w) images were acquired using a magnetization prepared rapid acquisition with gradient echo sequence, having the following parameters: repetition time = 1900 ms, echo time = 2.52 ms, flip angle = 80°, number of slices = 192, slice thickness = 1 mm, matrix = 256 × 256, and in-plane voxel resolution = 1 × 1 mm^2^. Rs-fMRI images were obtained using a gradient echo single-shot echo-planar image sequence with the following parameters: matrix = 64 × 64, repetition time = 1000 ms, echo time = 24 ms, in-plane resolution = 3.4 × 3.4 mm^2^, slice thickness = 3.4 mm, number of slices = 34 with no gap, slice orientation along AC–PC, and number of volumes = 480.

### 2.3. Physiological Signals Acquisition

Physiological signals were acquired during the MRI scan through a physiological signal measurement system installed in the MRI scanner, with photoplethysmography and a breathing belt.

### 2.4. Data Processing

The acquired data underwent the preprocessing steps of 3D motion correction and slice time correction before removal of the physiological noise. Physiological noises, including cardiac and respiration signals, were regressed from the preprocessed rs-fMRI data using the PNM algorithm based on the RETROICOR method in FSL [39]. After the removal of physiological noise, rs-fMRI data were further preprocessed by DPABI [40] with the following preprocessing steps. Head motion effects were removed with the Friston 24-parameter model and artifacts from the CSF signal. These functional images were smoothed with a 6-millimeter FWHM filter and co-registered with each corresponding structural image. Rs-fMR images were spatially normalized to a standard coordinate space and a Montreal Neurological Institute space to extract rs-fMRI time series from the predefined anatomical regions of AAL3 [41]. After normalization, the five metrics, FC (of the left postcentral gyrus as a seed because the area is the first ROI of the atlas; it is large, with its signal expected to be more stable for conceivable variations such as segmentation error, which can lead to functional connectivity with other areas affected by their variations.), fALFF (mfALFF), ReHo (mReHo), VMHC (zVMHC), and DC (zDC_PositiveWeightedSum) were calculated through DPABI processing.

The intraclass correlation coefficient (ICC) was calculated via a random sample of two raters (two scans in a session) and rating targets (18 repeated sessions without separation of subjects), in order to evaluate the reliability of repeated rs-fMRI measurements in a session [42,43]. For ICC evaluation, the values of the five repeated rs-fMRI metrics fALFF, ReHo, FC, VMHC, and DC were defined as raters, with the subjects defined as targets.

## 3. Results

**Figure 1:**
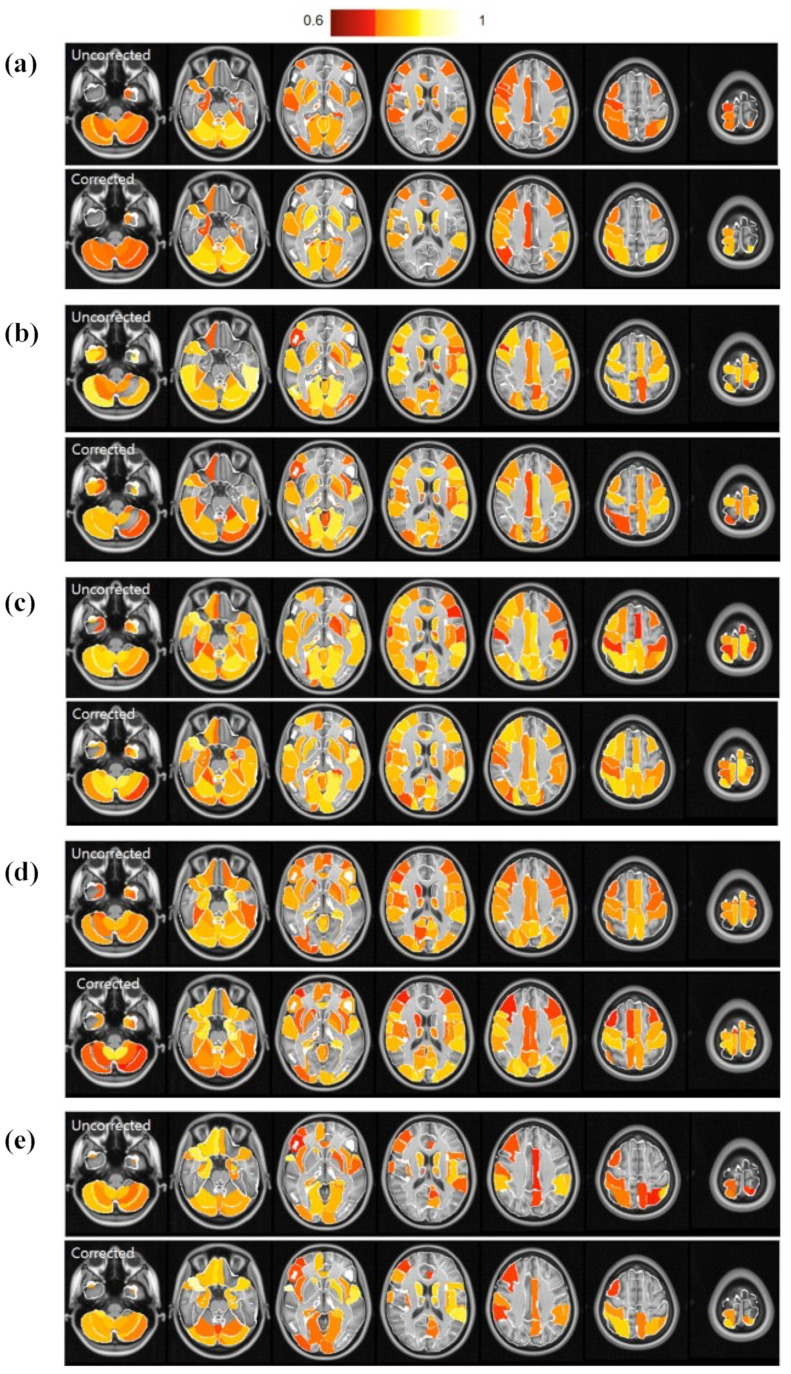
Maps of ICC values at representative ROIs based on AAL3, before and after the correction. (**a**) Functional connectivity (FC), (**b**) fractional amplitude of low-frequency fluctuation (fALFF), (**c**) regional homogeneity (ReHo), (**d**) voxel-mirrored homotopic connectivity (VMHC), and (**e**) degree centrality (DC).

**Figure 2:**
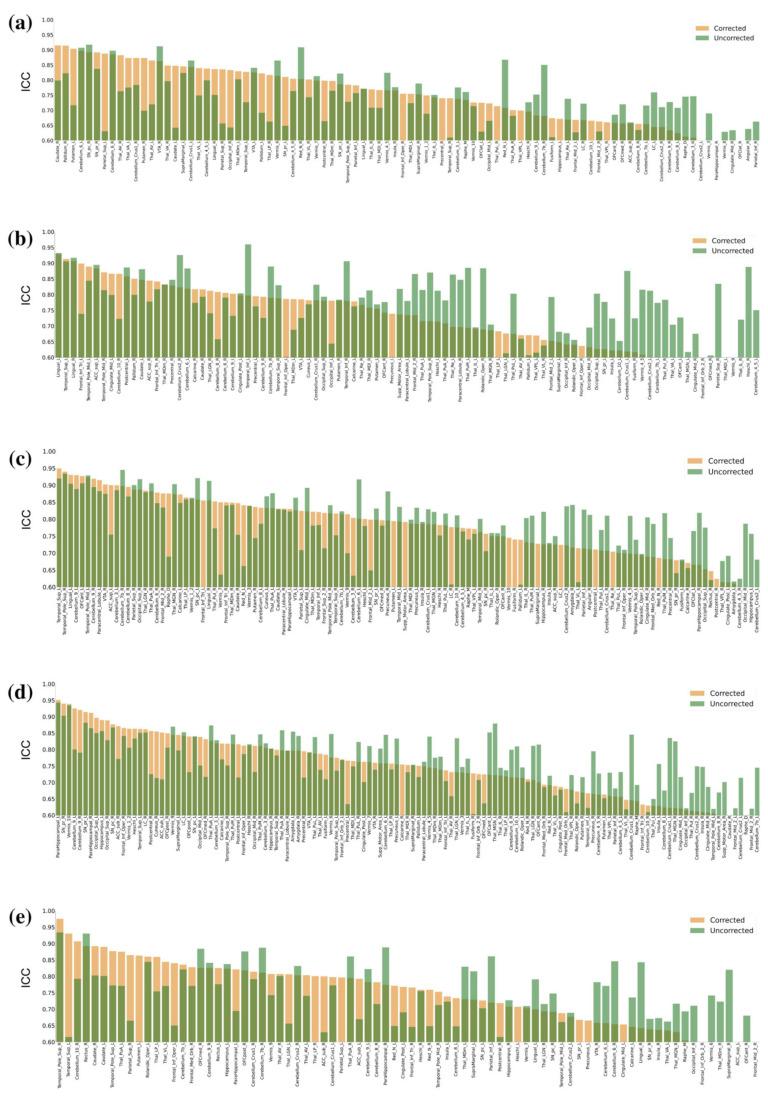
Plot of ICC values at all ROIs, corrected and uncorrected, through physiological noise removal. (**a**) Functional connectivity (FC), (**b**) fractional amplitude of low-frequency fluctuation (fALFF), (**c**) regional homogeneity (ReHo), (**d**) voxel-mirrored homotopic connectivity (VMHC), and (**e**) degree centrality (DC).

ICC values at individual ROIs (AAL3), before and after physiological signal removal, were compared to evaluate reliability. The maps show different effects of physiological signal removal. Some ROIs showed an improvement in reliability, while others did not (Figure 1). The ICC values of each ROI for each metric (fALFF, ReHo, FC, VMHC, DC) are shown in Figure 2.

**Figure 3:**
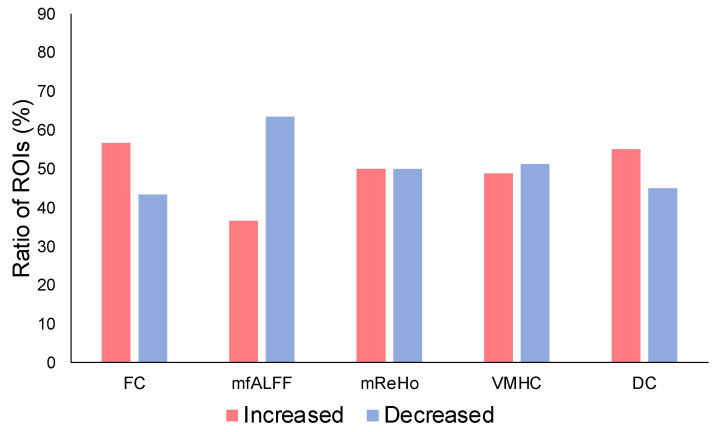
Ratio of ROIs with increased ICC values and with decreased values after the correction.

The ratio of ROIs with increased ICC values after physiological signal removal was evaluated for each metric modality (Figure 3). For FC, the ratio of ROIs with increased ICC values was within 56.67% while those with decreased ICCs were within 43.33%. The ratio of the ROIs with increased ICCs for fALFF was within 36.56%, and the ratio of the ROIs with decreased ICCs was within 63.44%. The ratio of the ROIs with increased ICCs for ReHo was within 50.00%, and the ratio of the ROIs with decreased ICCs was within 50.00%. The ratio of the ROIs with increased ICCs for VMHC was within 48.84%, and the ratio of the ROIs with decreased ICCs was within 51.16%. The ratio of the ROIs with increased ICC values for DC was within 55.00%, and the ratio of the ROIs with decreased ICC values was within 45.00%.

**Figure 4:**
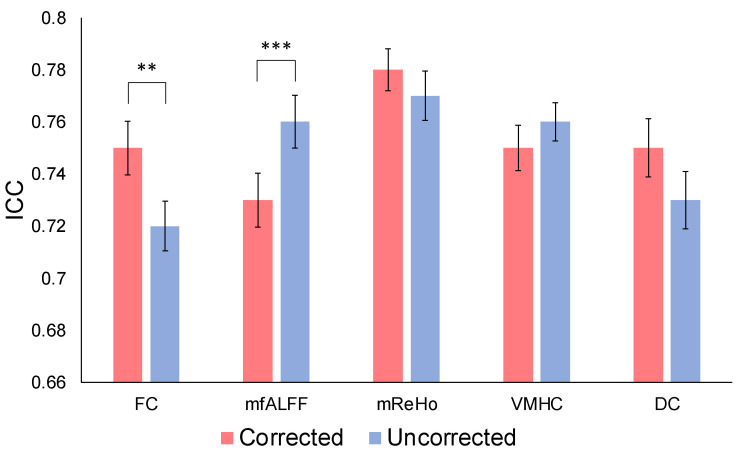
Comparison of mean ICC values over all ROIs, before and after correction, for functional connectivity (FC), fractional amplitude of low-frequency fluctuation (fALFF), regional homogeneity (ReHo), voxel-mirrored homotopic connectivity (VMHC), and degree centrality (DC). ** *p* < 0.01, *** *p* < 0.001 (paired *t*-test).

The mean ICC values, before and after correction, are shown in Figure 4. The mean ICC difference for all ROIs between the corrected and the uncorrected was significant for FC (paired *t*-test; *p* = 0.01), with an ICC increase of 3.5% after correction. The mean ICC value difference for all ROIs between the corrected and the uncorrected was significant for fALFF (paired *t*-test; *p* = 0.01), with an ICC decrease of 4.8%. The mean ICC differences for the other metrics, ReHo, VMHC, and DC, were not significant (paired *t*-test; *p* = 0.43, 0.41, 0.22, respectively).

Based on the effect of the correction, we compared the ratio of increased and decreased ICCs for FC when the difference value between before and after the correction was >0.1 (Table 1). ROIs of increased ICCs were 21.1%, and decreased by 7.7%. The increased ROIs included sub-regions of the thalamus, and bus regions of the striatum such as putamen, caudate nucleus, and pallidum, and the decreased ROIs mostly included sub-regions of the cerebellum and midbrain, etc.

## 4. Discussion

We examined the correction effect of rs-fMRI data by removal of physiological noise, evaluating the reliability of repeated measurements of the most-used rs-fMRI metrics. The present results demonstrate that the correction effect depended not only on the brain region, but the metric. Among the five metrics, the reliability in terms of the mean value of all ROIs significantly improved for FC, but deteriorated for fALFF; there were no significant differences for ReHo, VMHC, and DC. In addition, brain areas that showed ICC improvement or deterioration with the correction did not show any common trend in relation to their regional attributes of brain function (Table 1). Considering the dominance of ROIs with improved ICCs and the increase in mean ICC value, the correction was effective only for FC. 

Previous studies reported positive effects of physiological noise removal [26,28,44], showing that physiological correction improved rs-fMRI data stability, and produced better functional outcomes such as more specific activation maps, better correlation maps, and functional networks. With respect to stability, for example, a study showed that physiological correction could remove spurious activations in regions outside the gray matter shown in the uncorrected data [45]; another showed that physiological correction substantially reduced physiological noise components and increased temporal SNR [46]. In terms of functional outcomes, some studies showed that physiological noise correction increased the spatial extent, reduced apparent false positives in DMN [47], and increased DMN localization estimated by seed correlation to the seed region [48], leading to improved detection of consistent group differences [49]. Our finding of improvement in FC is consistent with those of previous observations.

For regional dependency of the correction effect, a study reported that respiratory-related signal fluctuations increased in cerebellar regions, with higher variances than for the primary motor cortex region [50]. Our data do not show consistent regional characteristics related to reliability with the previous study; that is, the physiological correction did not improve reliability more in the cerebellum than in the precentral region that corresponds to the primary motor cortex. 

For the dependency of the correction effect on the metrics, a previous study supports our observation that the correction effects vary depending on the metrics [51]. Our data demonstrate that whole-brain analyses can benefit from correction for FC metrics. 

For analyses based on specific ROIs or networks, a previous study showed that the physiological correction depended on the functional specialization of the brain region [52,53]. Our data were evaluated on brain regions based on anatomical segmentation. When computing ICC values and localizing functional areas, a further direction for the correction may be provided by the same evaluation. There are some limitations of this study. The regression model that was applied here may not reflect the dynamics of physiological sources on each voxel. Applying modeling dynamics at each voxel may improve the correction effects [54]. Furthermore, the sample size was small because we tried to focus on the evaluation of intra-session measurements by acquiring as much repetition data from the same subject as possible in a short period. We evaluated the data on the basis of rs-fMRI metrics, rather than using tSNR which may more directly reflect noise effects; many studies of rs-fMRI have been reported using these metrics.

Our results showed that the correction effect depends on the metric and brain regions, and do not make us confident about the physiological correction. However, a lot of rs-fMRI studies use FC as the metric and, in this case, the positive correction effect could be useful. In addition, our results may be informative for researchers who have taken rs-fMRI data without recording physiological signals, or for researchers do not have a facility for these measurements should they plan to use other metrics than FC; since physiological correction is not expected to further improve their results or conclusions, they could spare their efforts.

## 5. Conclusions

Rs-fMRI data correction by removal of cardiac and respiratory signals can improve the reliability of the FC metric during whole brain analysis; however, while some brain areas were improved, others deteriorated. The decision of whether to perform the correction should be based on the type of metric used.

## Figures and Tables

**Table 1:** ROIs of increased and decreased ICCs for each metric when the difference value between before and after the correction was >0.1.

Measure	Effect	AAL3 Brain Region
FC	Increased	Precentral_R
		Frontal_Inf_Oper_R
		Occipital_Inf_R
		Postcentral_R
		Parietal_Sup_L
		Parietal_Sup_R
		Caudate_L
		Caudate_R
		Putamen_L
		Putamen_R
		Pallidum_L
		Temporal_Sup_L
		Temporal_Sup_R
		Thal_AV_L
		Thal_AV_R
		Thal_LP_R
		Thal_VPL_L
		Thal_PuL_R
		SN_pr_L
	Decreased	Parietal_Inf_R
		Cerebellum_3_R
		Cerebellum_7b_R
		Red_N_L
		Red_N_R
		LC_L
		Raphe_D

## Data Availability

Not applicable.
